# Genomic epidemiology and molecular characterization of *Streptococcus pyogenes* isolates from pediatric infections in Beijing, China

**DOI:** 10.1128/spectrum.03914-25

**Published:** 2026-05-22

**Authors:** Zexuan Song, Lin Zhou, Wenjian Xu, Meng Tian, Li Yu, Xin Zhang, Ruixue Song, Jing Li, Lijuan Ma

**Affiliations:** 1 Department of Clinical Laboratory, Capital Center for Children's Health, Capital Medical University, Capital Institute of Pediatrics 12517, Beijing, China; Icahn School of Medicine at Mount Sinai, New York, New York, USA

**Keywords:** *Streptococcus pyogenes*, pediatric, antimicrobial resistance, emm1, emm12

## Abstract

*Streptococcus pyogenes* (Group A *Streptococcus*, GAS) remains a formidable global public health challenge. In this study, we conducted a high-resolution genomic epidemiology study on 176 non-invasive throat GAS isolates collected from pediatric patients in Beijing, China, between June 2024 and March 2025. Whole-genome sequencing was employed to characterize population structure, phylogenetic relationships, virulence genes, and antimicrobial resistance (AMR) determinants. The results showed that the population of GAS isolates in this study was dominated by emm12/ST36 (77.8%) and emm1 (22.2%) types. The emm1 isolates primarily belonged to ST1274 and ST28, with ST1274 being a single-locus variant of ST28. Crucially, the global M1_UK_ lineage was not detected. Phylogenomic analysis revealed that the emm12 population is structured into a dominant, conserved monophyletic clone (Clade A) co-circulating with diverse ancestral lineages. Pan-genome analysis further demonstrated an open genomic architecture characterized by a vast reservoir of accessory genes, indicating high evolutionary plasticity. While emm1 and emm12 exhibited distinct virulence signatures, a core virulence genome, including the the capsule-encoding *hasABC* operon, was universally conserved. Notably, we identified one emm12 isolate that acquired the emm1-associated superantigen *speA* gene via the ΦMGAS5005.1-like prophage. Furthermore, resistance to macrolides (97.7%) and tetracyclines (96.6%) was pervasive across both lineages, underscoring the severity of antimicrobial resistance in circulating GAS isolates. In conclusion, this study illuminates a distinct GAS epidemiological landscape in Beijing characterized by the local expansion of multidrug-resistant emm12 and emm1 clones. These findings emphasize the urgent need for continuous genomic surveillance to monitor the emergence of novel, hypervirulent recombinant variants within this distinct epidemiological context.

**IMPORTANCE:**

Group A *Streptococcus* poses a persistent global health challenge, capable of causing life-threatening invasive infections; thus, monitoring its evolving epidemiology is critical. Global surveillance activities have recently identified an upsurge of the hypervirulent M1_UK_ lineage, and our study of pediatric infections in Beijing identifies a distinct local trajectory dominated by multidrug-resistant emm12 and emm1 lineages. Notably, we documented a horizontal gene transfer event where a multidrug-resistant emm12 isolate acquired the *speA* superantigen gene—a virulence factor typically associated with the emm1 lineage. This finding illustrates that endemic clones possess the genomic plasticity to combine high virulence potential with existing antimicrobial resistance. Our work highlights that beyond monitoring global high-risk clones like M1_UK_, observing local evolutionary dynamics is essential to anticipate emerging regional threats.

## INTRODUCTION

*Streptococcus pyogenes* (Group A *Streptococcus*, GAS) remains a formidable global public health challenge, responsible for a significant burden of morbidity and mortality worldwide (1, 2). The clinical spectrum of GAS disease is wide and complex, ranging from common pharyngitis and scarlet fever to severe invasive infections, such as necrotizing fasciitis and streptococcal toxic shock syndrome (STSS), as well as serious immune-mediated sequelae, including acute rheumatic fever (ARF) (3, 4). Globally, GAS infections, particularly scarlet fever in pediatric populations, have undergone a concerning resurgence (5).

The global epidemiological landscape of GAS is dynamic, characterized by the emergence and expansion of specific clones defined by the emm genotype (6). While emm1 and emm12 have historically been the leading causes of both throat and skin infections (7, 8), a dramatic shift in GAS epidemiology has been observed in recent years (9). A number of high-income countries with well-developed healthcare systems, including Ireland, the United Kingdom, the Netherlands, France, Sweden, the United States, Australia, and New Zealand, reported a marked increase in GAS infections (7, 10, 11), particularly scarlet fever and invasive GAS (iGAS) hospitalizations compared with before the COVID-19 pandemic (12). In addition, this alarming trend has been largely attributed to the expansion of the M1_global_ genotype, specifically the hypervirulent M1_UK_ variant (13).

However, the epidemiological trajectory of GAS in China has historically diverged from this global trend, being predominantly characterized by endemic emm12 and a distinct M1_global_ sublineage (14). It is unknown whether the globally successful M1_UK_ clone has now breached this geographic barrier and begun to establish itself in China. Furthermore, the genomic characteristics of these established endemic clones—particularly their virulence arsenals and their response to sustained, region-specific antimicrobial selection pressure—remain largely uncharacterized. Moreover, the potential for horizontal gene transfer (HGT), which could equip highly transmissible endemic clones with novel virulence factors, represents an underappreciated but significant evolutionary threat that warrants investigation (15).

In this study, we conducted a high-resolution genomic epidemiology study of GAS isolates collected from pediatric patients in Beijing between 2024 and 2025. Herein, we characterize the population structure, virulence profiles, antimicrobial resistance determinants, and evolutionary context of these contemporary isolates. This study aims to provide a critical, up-to-date genomic snapshot of GAS in China and assess the local public health threat against the backdrop of a rapidly changing global landscape.

## MATERIALS AND METHODS

### Study design and bacterial isolates

This was a retrospective epidemiological study that included 176 clinical GAS isolates obtained from a tertiary teaching hospital affiliated with Capital Institute of Pediatrics between 1 June 2024 and 31 March 2025. The isolates were recovered from throat swab specimens collected from pediatric patients. These specimens were inoculated onto blood agar plates and incubated at 37°C overnight to obtain pure cultures. The metadata of the patient, including dates and demographic information, were retrospectively gathered from electronic medical records. Species identification for all GAS isolates was confirmed using matrix-assisted laser desorption/ionization-time of flight mass spectrometry (MALDI-TOF MS; bioMérieux, France), with *Escherichia coli* ATCC 8739 used as the quality control strain.

### Whole-genome sequencing

Genomic DNA was extracted using the Bacteria DNA Kit (TianGen, China) according to the manufacturer’s instructions, and the quality was assessed using Qubit 2.0. A pair-end library with an insert size of 500 bp was constructed and sequenced using an Illumina HiSeq X by PE150 strategy at the Annoroad Gene Technology company (Beijing, China). Raw reads were trimmed to remove adapter sequences and low-quality bases with Trimmomatic v0.39. The clean data were *de novo* assembled to generate draft genomes using SOAP denovo v2.04. The assembly quality was assessed with QUAST v5.0.2. Genomes were subsequently annotated using Prokka v1.14.0.

The 176 draft genomes exhibited a high degree of assembly contiguity, with sizes ranging from 1.78 to 1.91 Mb and N50 values between 97,803 and 263,330 bp. The number of contigs per assembly ranged from 39 to 86 (median = 60). These metrics, along with the identification of 1,666 to 1,866 predicted coding sequences (CDS) per genome, confirm that the assemblies are of sufficient quality for robust downstream comparative genomic analyses. Detailed assembly statistics and the accession numbers for all isolates provided in Table S1.

### Phylogenetic analysis

A core SNP alignment was produced by calling SNPs against reference genome MGAS9429 (NC_008021) with snippy v4.6.0 (https://github.com/tseemann/snippy) using default parameters. Recombination sites identified within this alignment were subsequently detected and masked using Gubbins v2.3.4. The resulting recombination-filtered core SNP alignment, comprising 2844 SNPs, was used to construct a maximum-likelihood (ML) phylogenetic tree in IQ-TREE v2.0.6. The final tree was visualized and annotated using the Interactive Tree of Life (iTOL) web server (https://itol.embl.de/). Genomes were annotated using Prokka, and the resulting GFF3 files were used as input for pan-genome analysis using the Roary pipeline (v3.12.0) (16), which identified 1,485 core genes.

### Genomic characteristics

The sequence types (STs) of isolates were determined from the assembled genomes using mlst v2.23.0 (https://github.com/tseemann/mlst). The emm types of the isolates were determined using emm-typer (https://github.com/MDU-PHL/emmtyper). To systematically identify the presence of the hypervirulent M1_UK_ variant in our study, all emm1 draft genomes were screened for the specific 27 lineage-defining core-genome single nucleotide polymorphisms (SNPs), as previously described (17). The antimicrobial resistance (AMR) genes were identified using Abricate v1.0.0 (https://github.com/tseemann/abricate) by screening against the CARD database with default settings (≥80% identity and ≥80% coverage). Virulence factor genes were similarly identified using Abricate against the VFDB database. Prophage regions within the draft assemblies were identified and evaluated for completeness using the PHASTEST web server (**https://phastest.ca/**).

### Statistical analysis

Statistical analyses were performed using SPSS software (Chicago, Illinois, USA). Differences between groups were detected using Chi-square test or Wilcoxon rank sum test. *P*-values less than 0.05 were considered statistically significant.

## RESULTS

### Epidemiological features of clinical isolates

A total of 176 pediatric patients with culture-confirmed GAS infections were included in this study, which comprised 114 males (64.8%) and 62 females (35.2%). The age distribution was remarkably similar between the emm1 and emm12 two groups. The median age for both emm1 and emm12 infected patients was 7.0 years (IQR: 6.0–8.0). Notably, all 176 cases included in this study were non-invasive infections, primarily presenting as scarlet fever and pharyngitis. All patients presented with uncomplicated manifestations, respiratory tract infection (manifesting as pharyngitis or tonsillitis without rash) was the most common presentation, accounting for 59.7% (105/176) of all cases. Scarlet fever, characterized by a typical rash, was documented in 43.2% (76/176) of the patients, and the statistical analysis revealed no significant association between the emm genotype and clinical symptoms (Table 1).

**TABLE 1:** The clinical characteristics of GAS isolates in this study

Basic information	Total (*n* = 176) *n* (%)	emm1 (*n* = 39) *n* (%)	emm12 (*n* = 137) *n* (%)	*P* value
Age (yrs)	7.0 (6.0–8.0)	7.0 (6.0–8.0)	7.0 (6.0–8.0)	0.758
Gender				
Male	114 (64.8)	21 (53.8%)	93 (67.9%)	0.106
Female	62 (35.2)	18 (46.2%)	44 (32.1%)	
Clinical presentation				
Respiratory symptoms	105 (59.7%)	23 (59.0%)	82 (59.9%)	0.920
Rash	76 (43.2%)	16 (41.0%)	60 (43.8%)	0.757

### The genetic diversity of GAS isolates

The population structure of the 176 GAS isolates in this study was highly clonal. Overall, we identified two major emm types (emm1 and emm12), which were further classified into 20 distinct emm subtypes (Table S1). The emm12 type was the most prevalent, accounting for 137 isolates (77.84%, 137/176), which were primarily represented by emm12.0 and related subtypes. The emm1 type accounted for the remaining 39 isolates (22.15%, 39/176), comprising emm1.0, emm1.22, and emm1.25. Using the MLST scheme, the isolates were assigned to three major sequence types (STs), including ST36, ST1274, and ST28. A strong association between emm type and ST was observed: all emm12 isolates (*n* = 137) belonged to ST36, and all emm1 isolates (*n* = 39) belonged to either ST1274 (*n* = 34) or ST28 (*n* = 5). This pattern highlights the stability of these major clonal complexes circulating among pediatric patients in Beijing.

### Pan-genome analysis

**Fig 1:**
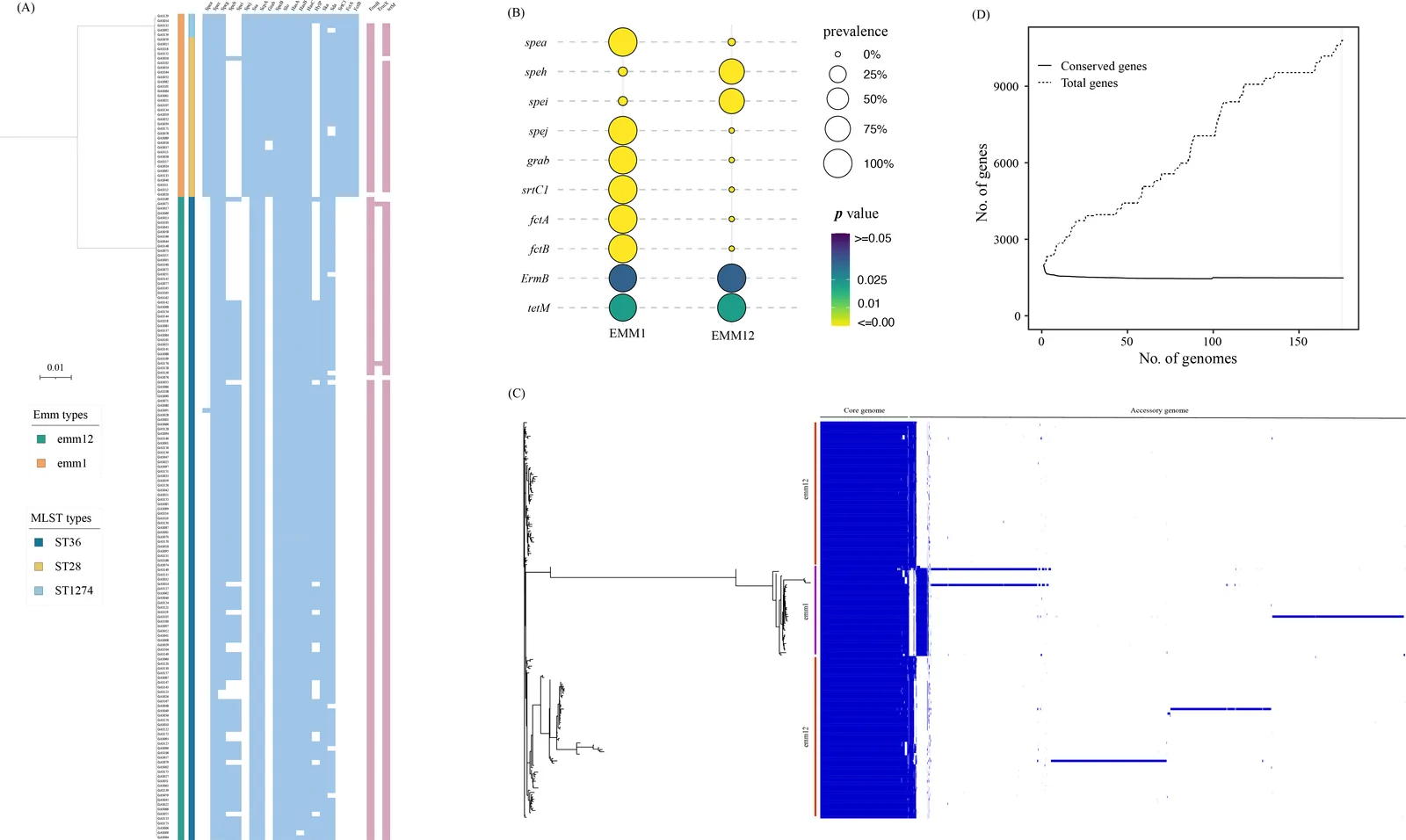
Genomic landscape and population structure of 176 GAS isolates in this study. (**A**) Maximum-likelihood phylogenetic tree based on core SNPs of 176 GAS isolates in this study. The major clades corresponding to emm1 and emm12 genotypes are highlighted. Tip annotations indicate the presence (color squares) or absence (white squares) of key virulence genes and antibiotic genes. (**B**) Correlation between emm genotypes and specific genes. The bubble size corresponds to gene prevalence (%), and the color intensity indicates the statistical significance of the distribution difference. (**C**) Pan-genome presence/absence matrix. The dark blue block represents the core genome (genes present in >99% of isolates), while the variable blue/white patterns represent the accessory genome. (**D**) Pan-genome accumulation curves. The solid line represents the number of conserved genes (core genome), while the dashed line tracks the total pan-genome size.

To evaluate the overall genomic diversity and plasticity of the circulating strains, we reconstructed the pan-genome of the 176 GAS isolates (Fig. 1C). A total of 10,780 unique gene clusters were identified across the cohort. The analysis revealed a “core genome” (present in >99% of isolates) consisting of 1,485 genes, which accounts for approximately 13.8% of the total gene pool. These conserved genes encode fundamental metabolic and cellular processes essential for GAS survival. Rarefaction curve analysis further demonstrated that while the core genome size stabilized rapidly, the total pan-genome size exhibited a continuous linear increase (Fig. 1D), indicative of an open pan-genome architecture. Importantly, this genomic plasticity is directly linked to specific evolutionary adaptations in our cohort. For instance, the accessory genome is enriched with integrative and conjugative elements (ICEs) harboring antibiotic resistance genes (e.g., *ermB*, *tetM*), reflecting adaptation to high clinical antibiotic selection pressure. Furthermore, the acquisition of prophage-associated virulence factors (e.g., *speA*) within specific lineages illustrates ongoing adaptation toward enhanced pathogenicity and niche expansion.

### Phylogenomic analysis of emm12 type

**Fig 2:**
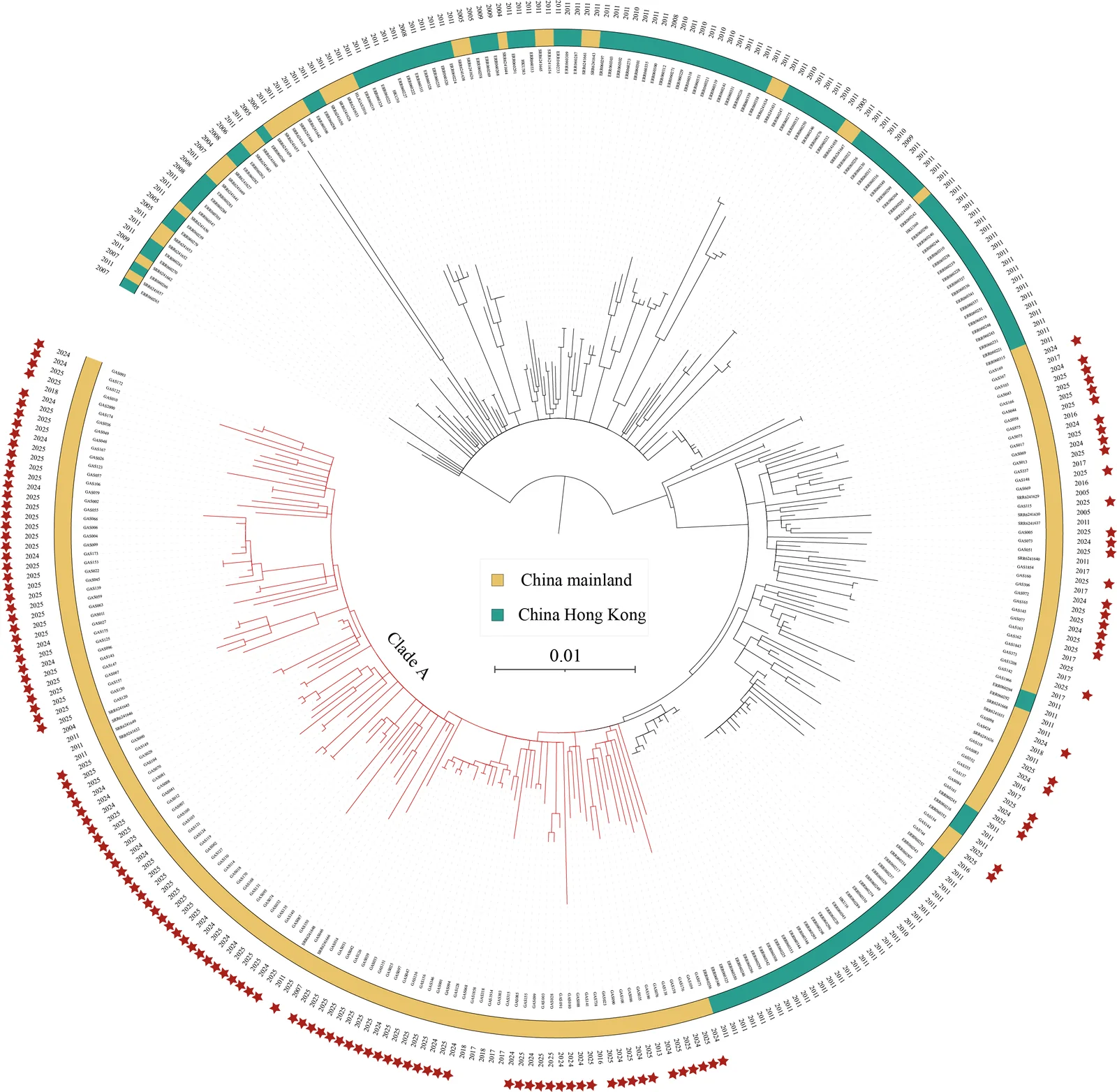
The phylogenetic tree was constructed based on core SNPs of 328 emm12 GAS isolates. The geographic location and date of isolation are shown on the tree (from inner to outer circles), according to the color legend. The red star shows the GAS isolates in this study.

To resolve the phylogenetic context of the dominant emm12 isolates from this study, a maximum-likelihood phylogeny tree was constructed using 137 emm12 isolates from this study and 200 emm12 genomes from NCBI (Table S2), including those from the 2011 Hong Kong scarlet fever outbreak and historical surveillance data from mainland China (18, 19). The resulting phylogeny revealed a striking phylogeographic structure (Fig. 2). We noted that a vast majority of our isolates (106/137, 77.4%) coalesced into a single, well-supported monophyletic clade (named Clade A), strongly suggesting a rapid clonal expansion event within the local region. In contrast, a notable subset of the local isolates (31/137, 22.6%) were phylogenetically dispersed, interspersed among diverse historical lineages from both Hong Kong and mainland China. Collectively, this characterizes the local emm12 population as being dominated by a recently expanded clone (Clade A), while simultaneously maintaining a reservoir of diverse, historically associated lineages.

### Superantigens and capsular genes

The virulence gene content of 176 GAS isolates was characterized (Fig. 1). The emm1 and emm12 isolates exhibited distinct virulence gene signature. The emm1 isolates displayed a remarkably uniform profile defined by the near-universal presence of a specific gene suite that was largely absent in emm12. Specifically, the superantigen genes *speA* (100% vs 0.7%) and *speJ* (100% vs 0%), the protein G-related alpha-2-macroglobulin-binding protein gene *grab* (94.87% vs 0%), and the FCT-3 type pilus-encoding cluster (*fctA*, *fctB*, *srtC1*; all 100% vs 0%) were hallmark features of the emm1 type. Conversely, the emm12 isolates were characterized by the high-frequency carriage of *sp*e*H* (75.2% vs 2.6%), *speI* (75.2% vs 2.6%), and *hylP* (75.2% vs 5.1%). The distribution of these genotype-specific genes was found to be highly statistically significant (*P* < 0.005).

In addition, a substantial set of virulence genes was highly conserved across all isolates, showing no statistically significant association with emm type (*P* > 0.05). These loci, including the genes encoding superantigens *spe*C and *speG*, streptolysin O (*slo*), streptokinase (*ska*), C5a peptidase (*scpA*), the cysteine protease *speB*, and the hyaluronic acid capsule synthesis operon (*hasABC*), were present in over 98% of all isolates analyzed, constituting a conserved core virulence repertoire among the predominant emm1 and emm12 lineages analyzed in this study.

Notably, one emm12/ST36 isolate presented an atypical profile (Fig. 1). Our analysis confirmed that this was the one isolate of emm 12 to harbor the superantigen *speA* gene. Analysis using the PHASTEST web server identified this region as an intact prophage, which sequence comparison revealed this prophage to be highly homologous to ΦMGAS5005.1, a mobile genetic element historically associated with the emm1 genotype. Crucially, sequence analysis confirmed that this ΦMGAS5005.1-like prophage was embedded within a large, continuous bacterial contig, and the *speA* gene was structurally verified to be located strictly within this delimited, chromosomally integrated prophage region, effectively ruling out sequencing artifacts. This indicates that the presence of *speA* in this emm12 background was mediated by a prophage-driven horizontal gene transfer event.

### Antimicrobial resistance genes

We investigated the distribution of key antimicrobial resistance genes associated with macrolide (*ermB*, *ermX*) and tetracycline (*tetM*) resistance. The EMM12/ST36 isolates displayed a remarkably homogenous and multidrug-resistant profile. Analysis confirmed that nearly all isolates within this lineage concurrently harbored *ermB* (99.3%, 136/137) and *tetM* (98.5%, 135/137). This conserved *ermB-tetM* profile was determined to be an essentially fixed characteristic of the emm12 population, predominately linked to the presence of ICE-emm12. However, we found that emm1 isolates also exhibited a strikingly high prevalence of resistance determinants. Despite historically being associated with lower resistance rates, the emm1 isolates in this study carried *ermB* and *tetM* at frequencies of 92.3% (36/39) and 89.7% (35/39), respectively. Similar to the EMM12 lineage, this resistance profile in EMM1 was strongly associated with the presence of ICE-HKU397 elements.

## DISCUSSION

This study provides a high-resolution genomic snapshot of GAS isolates circulating in Beijing, China, during 2024–2025, revealing the virulence and antibiotic characteristic of the predominant isolates. Our findings showed that the profound dichotomy between the two predominant genotypes, emm1 and emm12, which is consistent with the previous reports (9, 20, 21). These lineages exhibit not only mutually exclusive accessory virulence gene repertoires but also divergent evolutionary strategies in antimicrobial resistance, offering critical insights into the pathogenic potential and evolutionary trajectories of GAS in the region.

Consistent with the national surveillance data from China (18), our data showed that emm12/ST36 and emm1 (ST1274/ST28) represent the unequivocally dominant clones causing GAS infections among pediatric patients in Beijing. This distribution aligns with observations in pediatric populations in Shenzhen, China (20). The predominance of emm12, historically associated with pharyngitis, suggests it possesses high fitness for transmission and colonization within the current host population. In contrast, the less frequent but highly virulent emm1 clone persists in what may be considered a latent high-risk state, posing a continuous threat for sporadic, severe invasive disease. A key finding of our study is the conspicuous absence of the hypervirulent M1_UK_ variant. This stands in stark contrast to the epidemiological landscape in Europe, where the M1_UK_ clone has rapidly replaced M1_global_ and has now become the dominant emm1 type (7, 22). Our data powerfully reinforce the national-level observation by You et al. that M1_UK_ remains exceptionally rare in China (14). Therefore, while M1_UK_ is actively displacing other M1 clades globally, our results provide strong evidence that this successful lineage has not, as of yet, established a significant foothold in China. This localized pattern of classic, non-M1_UK_ emm1 driving non-invasive infections is strongly corroborated by a very recent report from Beijing (17). Future large-scale genomic surveillance will be crucial to fully elucidate regional adaptation and the evolutionary dynamics of these locally successful clones.

Our analysis highlights a pronounced divergence in the virulence gene repertoires between the emm1 and emm12 isolates, suggesting distinct pathogenic strategies. Despite this, a core virulence genome, including the *hasABC* operon, was universally conserved. A recent meta-analysis confirmed *hasA* is associated with invasive infection (23). However, as highlighted by Li et al. (24), most virulence-related factors, including the hyaluronic acid capsule operon, exhibit strong clonal linkage with specific emm types. Consequently, the pervasive presence of hasA in our non-invasive cohort does not necessarily imply a universally high baseline pathogenic potential. Instead, it more likely reflects the stable genetic conservation of this element within the predominant local lineages. Furthermore, any inherent genetic potential for severity is effectively mitigated by prompt medical intervention, as early diagnosis and antibiotic administration are highly effective at halting progression to invasive disease. This underscores the pivotal role of early clinical intervention in neutralizing the phenotypic expression of invasive genotypes.

Of particular concern, this study provides direct evidence of horizontal gene transfer of a critical virulence factor between disparate lineages, signaling the potential for a dangerous convergence of pathogenic traits. Specifically, our identification of an emm12 isolate acquiring *spea* gene—a potent, phage-encoded superantigen associated with severe disease—via the ΦMGAS5005.1. This finding demonstrates a key mechanism for genomic plasticity that can silently create novel, high-risk recombinant strains, thereby underscoring the urgent need for continuous genomic surveillance to monitor the frequency and spread of such variants.

Our study further underscores the severity of antimicrobial resistance in circulating GAS isolates. The macrolide- and tetracycline-resistant elements were found mostly in our GAS strains. These results are consistent with the previous findings of ICE-emm12 and ICE-HKU397 elements amongst multi-clonal emm12 strains of mainland China (18, 20). This severe resistance landscape is highly consistent with recent national surveillance data reported by You et al., which established that high-level macrolide resistance (>90%) has become a baseline characteristic of epidemic GAS in China (25). In stark contrast, contemporaneous data from the UK indicates a profoundly different epidemiological context, with only 24.3% of isolates reported to carry *tetM* and merely 4.9% carrying *ermA* (7). This discrepancy strongly suggests that intense, sustained, and localized selection pressure from macrolide and tetracycline use in China has acted as a powerful shaping force, posing a significant challenge to current empirical treatment guidelines.

Our study has several limitations. First, our cohort was collected from a single tertiary pediatric center in Beijing, which limits the generalizability of our findings to adults or other regions. Second, due to the retrospective nature of outpatient data collection, detailed clinical metadata were not always available, limiting granular genotype-phenotype correlations. Future studies with comprehensive clinical records are warranted to further investigate these associations. Finally, the cross-sectional design (2024–2025) and the absence of invasive isolates preclude the assessment of long-term temporal dynamics, such as determining the exact origin time (tMRCA) of the circulating clones through Bayesian evolutionary analysis, and direct comparisons between invasive and non-invasive strains.

In conclusion, our genomic investigation of pediatric GAS isolates in Beijing reveals an epidemiological landscape that diverges from the M1_UK_-driven resurgence reported in several countries. Instead of imported hypervirulent isolates, the region is facing a challenge characterized by the rapid clonal expansion of multidrug-resistant emm12 and emm1 lineages. The identification of an emm12 isolate that has acquired the potent superantigen *spea* via phage-mediated HGT highlights the ongoing potential for lineage convergence. Together with the intense antimicrobial selection pressure driving high-level resistance, these findings suggest that the pediatric population remains vulnerable to the emergence of novel recombinant strains. Consequently, continuous genomic surveillance is essential to monitor these evolutionary shifts and inform regional public health strategies.

## Data Availability

The genome sequences of the GAS isolates in this study have been deposited in the NCBI database under the accession numbers JBYEIV000000000 to JBYEPO000000000.

## SUPPLEMENTAL MATERIAL

Tables S1 and S2 spectrum.03914-25-s0001.xlsx Table S1: The detailed information of the 176 GAS isolates in this study. Table S2: The public genome of the emm12 isolates used in this study.

ASM does not own the copyrights to Supplemental Material that may be linked to, or accessed through, an article. The authors have granted ASM a non-exclusive, world-wide license to publish the Supplemental Material files. Please contact the corresponding author directly for reuse.
